# Exploring the knowledge gaps and reporting procedures for adverse events among professional nurses: evidence from Mopani district, Limpopo province, South Africa

**DOI:** 10.3389/frhs.2026.1951525

**Published:** 2026-09-24

**Authors:** Manyoga Blantina Mathoto, Tebogo Maria Mothiba, Masenyani Oupa Mbombi, Mxolisi Welcome Ngwenya, Livhuwani Muthelo

**Affiliations:** 1 Department of Nursing Science, University of Limpopo, Mankweng, South Africa; 2 Faculty of Health Sciences, University of Limpopo, Mankweng, South Africa

**Keywords:** adverse events, patient safety, patient safety incidents, professional nurses, reporting procedure

## Abstract

**Background:**

Adverse events refer to incidents or mistakes that cause harm to patients due to medical management, rather than being a result of the patient's illness or its complications. These events can occur at any point during the care process. Reporting adverse events is a key strategy in their prevention, and having a clear understanding of what qualifies as an adverse event, along with the correct reporting procedures, is essential for ensuring effective reporting practices.

**Aim:**

This study explored the extent to which professional nurses at selected public hospitals in Mopani District, Limpopo Province, South Africa understand adverse events and the reporting procedures of adverse events.

**Method:**

A qualitative exploratory-descriptive design was employed. Data were collected through in-depth, semi-structured interviews with 26 professional nurses, selected using purposive sampling. Data were analysed thematically using both deductive and Tesch's open coding method.

**Results:**

While most participants demonstrated awareness of adverse events and reporting processes, notable knowledge gaps were identified. Some participants did not understand what constitute an adverse event and conflated adverse events with near misses. Additionally, inconsistencies regarding reporting procedures, responsible personnel for reporting and reporting timelines were evident.

**Conclusion:**

The study revealed insufficient knowledge among professional nurses regarding adverse events, reporting procedures, and different types of patient safety incidents. To address these gaps, targeted educational interventions and reinforcement of standard operating procedures are recommended to enhance reporting practices and promote patient safety.

## 1 Introduction

Adverse events are a significant global challenge affecting patient safety within healthcare systems. The National Guidelines for Patient Safety Incidents Reporting and Learning in the Public Sector of South Africa describe adverse events as incidents or errors that result in harm to a patient that is related to medical management, in contrast to disease complications or underlying disease (1). These incidents include, but are not limited to, medication and equipment errors, patient falls, pressure sores, hospital-acquired infections, and inefficiencies in making a clinical decision (2). Adverse events affect patients at all stages of care, in both acute and outpatient settings, and they are transversal globally (3). Associated with these adverse events are consequences such as disability, unnecessary medical costs, and psychological, emotional, family, and societal impacts (4).

All healthcare providers take an oath to “Do no harm”, despite their best efforts, healthcare providers still experience adverse event occurrences in the healthcare setting (5). This is because the health care delivery systems are complex by design and prone to errors (6). Hence, most health care organisations have come to terms with the fact that adverse events are inevitable (7). Patient harm due to adverse events has been reported to be one of the top 10 causes of death and disability in the world (6). In the United States (US), over 250,000 patients will experience an adverse event while receiving hospital care each year (8). In low and middle-income countries, hospitals, available evidence suggested that 134 million adverse events occur due to unsafe care (9).

In sub-Saharan Africa, 30% of deaths are attributed to adverse events (10). In South Africa, the Gauteng Department of Health released statistics of adverse events in 2018. The statistics showed that over 2016, 2017 and 2018, the number of patients who had experienced severe adverse events was 20,417 (11). Mgobozi and Mohamed's (12) study findings showed 4.12 patient safety incidents per 10,000 inpatient days in KwaZulu-Natal.

Literature indicates that 50%–70% of adverse events are preventable (13–15). One of the main strategies in managing and preventing adverse events is to report them (16). Thus, successful process of adverse events prevention cannot be implemented without reporting them (17). Through adverse events reporting, organisations can learn from previous mistakes and, of course, hone the organization's ability to identify risk factors early to prevent or reduce incidents (18).

According to the knowledge, attitudes, and practices model, sufficient knowledge fosters attitudes and beliefs, which in turn propel reporting practices and behaviours (19). Previous studies have identified the knowledge of what constitute an adverse event, reporting protocol and procedure, importance of reporting, providing of information about patient safety incidents as factors associated with the reporting behaviour of healthcare professional (20–23). Thus, adverse events reporting intentions and behaviours require nurses to be empowered with information about adverse events, the reporting systems and procedures in healthcare institutions (19).

Numerous studies showed that underreporting of adverse events persists worldwide (19, 23–26). However, research studies on the knowledge and understanding of adverse events and the reporting procedure among professional nurses based on the South African context are limited in the literature. Local studies focused on analysis of adverse events in healthcare facilities, epidemiology of patient safety incidents and the experiences of healthcare providers following adverse events (5, 12, 24, 27, 28). As it is indicated in the literature that adverse events reporting promotes patient safety, it is necessary to have a better understanding of nurses’ knowledge and understanding of what an adverse event is and the importance of adverse events reporting and the reporting procedure. The aim of this study was therefore to explore the extent to which professional nurses at selected public hospitals in Mopani District, Limpopo Province, South Africa understand adverse events and the reporting procedures of adverse events. The findings of the results will undoubtedly help to improve comprehension of patient safety incidents, allow for clarification, and encourage the reporting of adverse events, thereby enhancing patient safety.

## 2 Research method and design

### 2.1 Study design

A qualitative explorative descriptive design was employed to explore the extent to which professional nurses at the selected public hospitals understand adverse events and the reporting procedures of adverse events. The primary reason the authors chose to employ a qualitative study design was because qualitative research approaches are open ended and can help reveal themes that could not be revealed by quantitative approaches (29).

### 2.2 Study setting

The study was conducted at the selected public hospitals in Mopani District, Limpopo Province. Limpopo Province is situated in the Northeast of South Africa. Out of the eight public hospitals in Mopani district, all the public hospitals located in the Greater Tzaneen Municipality were selected for this study. The Greater Tzaneen Municipality has three public hospitals. The names of the public hospitals selected for this study are referred to as Hospital A, Hospital B, and Hospital C to ensure anonymity. Hospital A is the only regional hospital in the Mopani district that is utilised as a referral hospital by all the public hospitals in Mopani district. The reason all the hospitals in Greater Tzaneen Municipality were chosen for this study is that all of them use the same reporting systems and provide the same comprehensive and specialised medical services.

### 2.3 Sampling method

The study employed Convenience and non-probability purposive sampling methods to select the participants. Convenience sampling is a type of sampling that is characterised by choosing participants based on their closeness to the researcher (30). The use of this sampling method allowed the authors to collect data from the respondents who met the inclusion criteria and were readily available in the wards at the time of data collection.

### 2.4 Recruitment of participants

The participants of this study were professional nurses employed at the three selected public hospitals, as they are expected to have knowledge and understanding of adverse events and are responsible for management of adverse events. The inclusion criteria included professional nurses who were permanently employed for more than a year because it is believed that they are familiar with the standard operating procedures, policies and guidelines at their workplace.

After obtaining the required approval to carry out the research study, the authors visited all the wards in the three selected public hospitals to explain to the professional nurses and their operational managers about the study's purpose, objectives and its significance. The participants were also informed that the research project is for academic purposes only and that the results of the study will be used to come up with ways to promote the reporting of adverse events. The authors highlighted to the participants how the interviews would be conducted. Both the authors and participants agreed on the days and times the interviews will be conducted. The participants indicated that the convenient time for interviews will be during visiting hours.

### 2.5 Data collection

Data were collected over three months from September 2024 until November 2024 by the first author through one-on-one in-depth interviews with professional nurses and nurse managers who agreed to participate. Semi-structured interview questions were used to ensure that all participants get the same questions in the same order and in the same manner. The interview guide was developed based on the literature reviewed for this study.

The first author started the interviews at hospital A until no new data was obtained at participant number 19. Then, after that the author moved to hospital B, where four participants were interviewed and no new data emerged. However, the author continued to hospital C to see if any new data were emerging and two additional interviews were conducted and no new data emerged. Therefore, the total number of participants interviewed is 26 and data saturation was achieved at participant number 19. Prolonged engagement was employed during data collection. Thus, the interviewer (first author) spent extended period of time with the participants to obtain rich and thick data. The rich and thick data provided the author with more information about the extent to which professional nurses understand adverse events and the reporting procedures.

### 2.6 Data collection procedure

The participants were approached in the wards and brief information about the study and its purpose was provided to the professional nurses who were available in the wards during the data collection period. Those who agreed to participate in the study were requested to sign consent forms and fill in a questionnaire for demographic data. The questionnaire consisted of the following variables: age, gender, highest level of qualification, category of professional nurse (e.g., general nurse, specialist nurse or nurse manager), years of experience as a professional nurse, and type of working unit. To prevent disturbances of daily routine in the wards, the researcher was offered an office by nurse managers in each ward.

Before being interviewed, the participants were informed that their responses would be audio recorded to prevent data loss. It was also made clear to the participants that all information gathered will be kept private and that all ethical guidelines will be adhered to during the entire interview session. The interviews were conducted in English and they lasted from 30 min to 45 min.

The interview guide used by the interviewer was as follows:

“Could you please tell me what an adverse event is”? You are allowed to give me examples if you want to.

“Could please tell me how you report adverse events in this unit or hospital”?

Depending on each participant's response, follow-up questions were posed. Field and observational notes were gathered. To get more detailed information and clarification from the participants, probing was employed.

### 2.7 Data analysis

After recording and transcribing interviews the researchers first analysed the data through deductive thematic analysis. The deductive thematic approach comprises examining data based on literature review or pre-existing themes (31). The first author read and evaluated the data from the transcribed interviews and after that the author revisited the research questions and objectives of the study. From the research questions, objectives of the study and the data on the transcribed interviews the author expected to discover the following two themes: Professional nurses’ knowledge and understanding of adverse events and Professional nurses’ knowledge and understanding of the procedure of reporting adverse events. These themes were predetermined based on the knowledge, attitudes, and practices theory (19). This theory states that knowledge is the predicate and basis of behavioural intention, meaning that sufficient knowledge fosters attitudes and beliefs, which in turn propel reporting practices and behaviours (19).

In order to identify codes or statements that could be linked to the predetermined themes the eight (08) steps of Tesch's open coding method of qualitative data analysis were utilized. The analysed data were then discussed amongst the authors and after that it was submitted to an independent coder who also used eight (08) steps of Tesch's open coding method of qualitative data analysis. The process on how the eight (08) steps of Tesch's open coding method of qualitative data analysis was used to formulate themes is described below:

Step 1: Reading through the data
  The authors repeatedly listened to the audio-recorded data to become acquainted with it. The data was then transcribed verbatim and all the verbatim transcripts were read multiple times and then the meaning of the data emerged and whilst reading some ideas came and they were written down as well as the emerged data.
Step 2: Reduction of the collected data
  Following familiarisation with the data, the authors reduced the amount of data gathered by generating codes according to the frequency or presence of concepts in the verbatim transcripts. The authors conducted manual line by line coding to identify codes or topics that were relevant to the research questions. Then a list of all codes was compiled.
Step 3: Asking questions about the meaning of the collected data
  The topics or codes that were linked to one another were grouped together, while unrelated topics were clustered separately. In order to obtain the meaning of the data collected, the authors thoroughly examined all the topics that emerged.
Step 4: Abbreviation of topics to codes
  The topics were then abbreviated as codes and the codes were written next to a fitting segments of the text to see if new categories and codes emerged. In order to differentiate the codes, all significant instances of the data associated with a particular code were included.
Step 5: Development of themes and sub-themes.
  The authors then generated themes and sub-themes using coded data and the related texts. These themes were examined to ensure they aligned with both the coded extracts and the entire dataset. In the process of reducing the list of themes, the ones that were associated were compacted together.
Step 6: Comparison of the codes, topics, and themes for duplication
  Re-analysing of data was done to check for duplication of codes and themes, and also to check if it is necessary to refine topics, codes and themes. The researcher went over the list of all codes again and during this process some were recoded and others were grouped together so that they suit the description.
Step 7: Initial grouping of all themes and sub-themes
  The data for each theme were compiled in a single column and preliminary analysis was performed.
Step 8: Validating the accuracy of the information
  After the data for each theme were compiled in a single column and performing preliminary analysis the authors and independent coder discussed the themes that were separately developed and they all the agreed on the final themes and sub-themes.

### 2.8 Measures to ensure trustworthiness

The following principles of trustworthiness were adhered to: credibility, confirmability, dependability, and transferability. Credibility was ensured by in-depth one-on-one interviews and data from observational and field notes were used. Also, the interviewer spent enough time in the field with the participants and that enhanced the gathering of rich and thick data that provided more information about the participants’ knowledge and understanding of adverse events. Direct quotations were used to validate the accuracy of the data from the interviewees. To ensure that the researcher's preconceived ideas do not have influence during data collection, analysis and interpretation, personal views and preconceived ideas were all written down and set aside before commencing data collection. To ensure that different viewpoints were taken into account peer debriefing with co-authors was utilised. Throughout the research process the researcher tried to be neutral.

To lessen the impact of personal bias, reflective notes were systematically cross-checked against interview transcripts. Dependability and confirmability were ensured by facilitating discussions among all the authors regarding the development and comparison of themes, using an audit trail of the verbatim transcripts and audiotapes to identify and categorise the codes, as well as facilitating a discussion between the authors and independent coder to the findings of the study. Transferability was ensured by opting for purposive sampling of participants, outlining the study setting, data gathering procedure and utilising a variety of data sources. To increase the validity of the research findings, a variety of literature sources were consulted, including books, papers, and Internet searches (32).

### 2.9 Ethical considerations

Approval to conduct the study was obtained before the study commenced from Turfloop Research Ethics Committee (TREC) of the University of Limpopo (clearance number TREC/45/2024: PG). Authorization to gather data at the three selected public hospitals was granted by the Limpopo Department of Health (Reference number: LP 2024-04-018), the Mopani district manager and the Chief Executive Officers (CEO's) of the selected public hospitals. In adherence to anonymity and confidentiality code numbers were used instead of capturing the real names of the participants and three selected hospitals. The authors made sure that the questionnaires are free of any data that could be used to identify the participants. The written consent prior to collection of data was obtained from each participant. The participants were informed about their right to withdraw from the study at any time, even if they had initially agreed to participate. In adherence to the right to protection from exploitation the participants were given the reassurance that their involvement or any information they may supply to the researcher won't be utilized against them in any way. To ensure privacy the participants were interviewed in a private office in the wards of the hospital under study.

## 3 Results

### 3.1 Demographic profile of the participants

The study consisted of 26 participants, twenty-two of whom were females (85%) and four were males (15%). Regarding the participants’ age range, the majority of them were in the age group of 21–30 (34,6%) and 51–60 (34,6%). Thirteen (50%) of the participants had a diploma as the highest level of education. The participants were grouped into three categories of professional nurses, and out of the 26 participants, eleven (42%) were in the category of general professional nurses, seven (27%) were specialist professional nurses and eight (31%) were nurse managers. Regarding years of experience as a professional nurse, the majority of the participants (38%) were in the category of 16 years and above. Regarding the type of unit (ward) the participants were allocated to, the majority of them were in the medical (19%) and Intensive Care Unit (19%). The demographic profile of the participants is shown in Table 1.

**Table 1:** Demographic profile of the participants.

Characteristics	Frequency	Percentage
Gender Males Females	4 22	15% 85%
Age 21–30 31–40 41–50 51–60 61–65	1 9 6 9 1	3.8% 34.6% 23% 34.6% 3.8%
Highest level of education Diploma Postgraduate diploma Bachelors’ degree Masters	13 6 6 1	50% 23% 23% 3.8%
Category of professional nurse General professional nurse Specialist professional nurse Nurse manager	11 7 8	42% 27% 31%
Years of experience 1–5 6–10 11–15 16 years and above	2 8 6 10	8% 31% 23% 38%
Type of working unit Medical ICU Maternity Gynaecology Paediatric Theatre Casualty Out-patient Orthopaedic Combined disciplines (Medical, surgical) Occupational Health and Safety	5 5 1 2 1 2 3 1 3 2 1	19% 19% 3.8% 7.7% 3.8% 7.7% 12% 3.8% 12% 7.7% 3.8%

### 3.2 Themes and subthemes

The two key themes that emerged from the data were professional nurses’ knowledge and understanding of adverse events and professional nurses’ knowledge and understanding of the procedure of reporting adverse events. Eight sub-themes were also identified. The key themes and subthemes are shown in Table 2.

**Table 2:** The key themes and sub-themes identified from the data.

Themes	Subthemes
1. Professional nurses’ knowledge and understanding of adverse events	1.1 Definition of adverse event and examples
	1.2 Misunderstanding of what constitutes an adverse event.
2. Professional nurses’ knowledge and understanding of the procedure for reporting adverse events	2.1 Reporting adverse events by writing on an adverse event form.
	2.2 Reporting adverse events by writing in the adverse event book.
	2.3 Reporting lines of adverse events.
	2.4 Persons responsible for reporting and recording adverse events.
	2.5 Timelines and guidelines for reporting.
	2.6 Escalation of adverse events and adverse event reports to the quality assurance office/manager.

#### 3.2.1 Theme 1: professional nurses’ knowledge of adverse events

Some participants demonstrated knowledge and understanding of what an adverse event is. However, the responses of other participants showed that though they have knowledge and understanding of what an adverse event is, they have a misunderstanding of what exactly constitutes an adverse event.

##### 3.2.1.1 Sub-theme 1.1 definition of an adverse event and the examples

The participant could not give the exact or official definition of an adverse event, but they were able to give examples of adverse events. However, some responses provided were satisfactory, and others were not.

Satisfactory responses were as follows:

**Participant PN3 (General professional nurse): “**An Adverse event is the event that can occur whilst working with a patient. Let's say maybe I'm busy working with the patient, and I'm having an object like a pair of scissors or something like that. I happened to injure the patient by mistake, and the patient got injured, I cut the patient with that scissors by mistake, and the patient had to be sutured”.
**Participant PN 14** (**Specialist professional nurse)**: “An adverse event is a situation whereby an incident occurs at the workplace. Like maybe while you are trying to administer a prescribed treatment, you find that due to the business of the unit, you administer the prescribed treatment to the wrong patient. So, that can be taken as an adverse event”.
**Participant PN7 (Nurse manager): “**According to me, simple words for an adverse event is something that has occurred in the unit, which is causing harm to the patient and also affecting the department. Sometimes the department will be sued because of that thing if we have failed to inform them, but even if we informed the department, the affected person can come and sue the department”.

Responses that were not satisfactory: (quotations in bold letters indicate unsatisfactory)

**Participant PN10 (General professional nurse): “**I think **it's any incident that happens in the ward**. For example, maybe a patient falling from the bed or giving the wrong treatment to the patient”.
**Participant PN3 (General professional nurse**): “**According to me, adverse events are being misinterpreted because, you must report something that has not happened. I am relating to a near falling incident”**
**Participant PN16** (**Nurse manager):** “An adverse is an occurrence that happens of which it was unprepared and unforeseen**. It can be related to the patient's injuries or illness.** So, it is an unforeseen circumstance that can result in harm of a patient or a client, or even the staff themselves”.
**Participant PN 8 (Nurse Manager):** “An adverse event is anything that is deviating from a normal situation or a normal standard of doing things. For example, a patient can come to theatre for caesarean section, but at the end of the operation you find that patient is having burns wound caused by Diathermy or **if the nurses or doctors go into a physical fight or maybe if they're insulting each other, it's an adverse event, because it is deviation from a normal situation”.**

##### 3.2.1.2 Sub-theme 1.2: misunderstanding of what constitutes an adverse event

The findings of the study revealed that some participants had a misunderstanding of what constitutes an adverse event. Some confused adverse events with other types of patient safety incidents and others provided incorrect description. For example, PN2 and PN 16 confused adverse events with near miss: The following quotations in bold letters indicate a misunderstanding of what an adverse event is:

**PN2 (Specialist professional nurse): “**It is any happening or occurrence which was not planned, and which may lead to or cause harm to the patient or injure the patient. In severe cases, it can even be fatal, causing death**. But some can be like maybe harmless, like for example near miss. So it must be reported. It's an adverse event, a near miss. Near miss is a type of adverse event. According to me, a near miss is a type of adverse event. The word adverse event is an umbrella name. So we have got different types of adverse events and amongst them is a near miss, which I'm always referring to”.**
**Participant PN16 (Nurse manager). Near miss is an adverse event related to falls, for example, when the patient nearly fell down, that is considered a near miss. So a near miss is also part and parcel of adverse event. The word is used only to categorize adverse event of falls.**

#### 3.2.2 Theme 2. professional nurses’ knowledge of the procedure of reporting adverse events

All employees in healthcare facilities have to report and document all adverse events (1). The authors sought to find out if participants working in the three selected public hospitals know and understand the reporting procedure of adverse events. The study revealed the participants’ differing knowledge regarding the documentation of adverse events report, the timelines and person responsible for reporting.

##### 3.2.2.1 Sub-theme 2.1: reporting adverse events by writing on an adverse event form

All adverse events should be recorded on a patient safety incident reporting form (1). Some participants indicated that when an adverse event has occurred in their work area or ward, they report by filling in the adverse event form:

**Participant PN3 (General professional nurse):** The manager or the supervisor must give me the form to write the adverse event. I must write how it happened, what time, things like that and what happened to the patient. I record everything on the form. Then I sign everything that I'm the one who report.

##### 3.2.2.2 Sub-theme 2.2 reporting adverse events by writing in the adverse event book

It is stipulated in the National Guideline for Patient Safety Incident Reporting and Learning in the Health Sector of South Africa—Version 2 that all healthcare institutions should have registers to keep statistical data on patient safety incidents (1). The guideline further explains that the register can be in the form of a book or separate pages kept in a file that is marked that it contains patient safety incident registers (1). However, the study findings revealed that some participants do not have a clear understanding of what the adverse event book is used for hence they reported that they report adverse events by writing in an adverse event book. Below are the quotes of the participants:

**Participant PN4 (Nurse manager): “**They write in the book. We have a book that is just talking to the date, time, what happened, and who was involved. So I'm expecting them to report and to record. Then from there I take it from the very same book, I'll be writing, including everyone who was involved, just to give me an account of what happened, exactly word by word, until they did the last action to make sure that everybody knows”.
**Participant PN9 (General professional nurse):** “If maybe the patient fell, I must write the incident in the incident book”.
**Participant PN18 (General professional nurse):** “You start verbally, followed by writing a statement in an adverse event book”.

##### 3.2.2.3 Sub-theme 2.3 reporting lines of adverse events

When the participants narrated how they report adverse events, they further explained to whom do they report adverse events to and the reporting line was also explained. The professional nurses report adverse events to their supervisors or the nurse manager (matron) of the ward, whereas the nurse managers report to the top management of the institution. The following statements describe the reporting line of adverse events:

**Participant PN1 (nurse manager**): “We report to the quality assurance manager, the CEO, and the deputy nursing manager. Normally, we call them. We inform them telephonically. The operational manager or whoever is in charge must report to the office of the quality manager”.
**Participant PN5 (General Professional nurse):** “During the week we report to our matron, the nurse manager in the ward and on weekends we report to the matron on call”.

##### 3.2.2.4 Sub-theme 2.4: persons responsible for completing (filling in) the adverse events form

The study revealed the participants’ differing knowledge regarding the person responsible for filling in the adverse events form. The participants shared different information regarding the person responsible for recording adverse events on the adverse event form. This is evidenced by following statements:

**Participant PN4 (Nurse manager):** “The respective unit manager must take the form and complete it, starting from the first line until the last line. But reporting, anybody can report. Filling the form is the unit manager's responsibility.”
**Participant PN7 (Nurse manager): “**My subordinate, the professional nurse, must know how to fill the form because sometimes I will not be around. So they should not say, we won't submit the form because our manager is not around. The professional nurse is the one that will fill the document, even if I'm not around”.
**Participant PN 14 (Specialist professional nurse): “**Yes, those who are enrolled nurses and assistant nurses they are only allowed to just fill in the statement but not to complete the whole form. On the form there is a part where other categories are not allowed to fill in. And that is the practice which I don't understand, because now it's like when an incident occur only the supervisor or professional nurse in-charge of that day must be the one to fill that form. But I take it as a culture that when something in the ward happens the supervisor or the manager of the unit must be the one to report it to the superiors”.

##### 3.2.2.5 Sub-theme 2.5: timelines and guidelines for reporting

The timeline for reporting adverse events depends on the severity assessment code (SAC) of the incidents. There are four classes in the SAC: classes 1, 2, 3, and 4. SAC 1 is when serious harm or death, SAC 2 is for incidents that caused moderate harm, SAC 3 is for incidents that caused minor harm, and SAC 4 is for incidents that caused no harm (1). According to the National Guideline for Patient Safety Incident Reporting and Learning in the Health Sector of South Africa—Version 2, all SAC 1 incidents should be reported within 24hrs (1). This implies that adverse events that falls into the classification of SAC 1 should be reported within 24 h. However, the study revealed that participants’ knowledge regarding the timelines for reporting adverse is divergent and this is supported by the following statements:

**Participant PN 2 (Specialist Professional nurse): “**We report within 24 h, that's how we report adverse events”.
**Participant PN4 (Nurse manager):** “The guideline gives us principles on what is it that we need to do. Immediately, you identify that there is an adverse event. It needs to be reported within 72 h”.

##### 3.2.2.6 Sub-theme 2.6: escalation of adverse events and adverse event reports to the quality assurance office/ manager

The findings revealed that some of the participants know where the completed adverse event forms are submitted to, whereas some participants reported that they are not sure. Below are the quotes from the responses of the participants:

**Participant PN13 (Specialist professional nurse):** “After completing the form, we submit it to the operational manager and she has a part where she is supposed to complete and the form will be taken to quality section so that they can review the incidents”.
**Participant PN3 (General professional nurse):** “The form goes to the manager in the ward. The manager is then going to escalate it and report to wherever she or he is reporting to. I don't know where she is taking the form or where she is reporting to. I'm not sure. Maybe it's the quality office or somewhere I am not sure”.
**Participant PN11 (General professional nurse):** “Our matron takes it to the form to the office up there, I am not sure of the office, but I think it is a quality department or office, something like that”.

## 4 Discussion

To our knowledge, this is the first qualitative study that examines professional nurses’ awareness and understanding of adverse events and how they are reported in the three selected public hospitals in the Mopani district, Limpopo Province, South Africa. The study explored the extent to which professional understand adverse events and the reporting procedures of adverse events. Knowledge of what constitutes an adverse event as well as the reporting procedure is a prerequisite for enhancing effective adverse event reporting amongst healthcare professionals (33). The present study revealed that though participants have knowledge of what an adverse event is and the reporting procedure of these events, some gaps in their knowledge were identified. These gaps could lead poor implementation of the national guideline of patient safety incidents reporting at the studied hospitals and these could affect safety of patients.

The majority of the participants in this study have understanding that an adverse event is an incident that results in harm to the patient. However, when describing or defining it they included the term near-miss, which is another type of patient safety incidents. This finding indicates lack of knowledge or misunderstanding of different types of patient safety incidents. Healthcare professionals’ lack of knowledge to recognize and perceive what constitutes an adverse event was also reported in previous studies (19, 23, 26, 28, 29, 32). It is worth noting that in the present study, specialist professional nurses and nurse managers are amongst participants who showed gaps in their knowledge of what constitutes an adverse event. The explanation of this finding could be that the nurses managers and specialist nurses are not giving the national guidelines for patient safety incidents reporting proper consideration and attention. This is of concern because specialist these professional nurses are thought to be responsible for encouraging other nursing categories to report adverse events.

Inadequate knowledge and misunderstanding of what constitute an adverse event and different types of patient safety incidents could lead to certain adverse events to be not recognized as such and that could compromise the safety of patients more so because some participants in this study indicated that it is not necessary to report near miss. A near miss is an incident that did not reach the patient but has a potential to cause harm (6). However, WHO (6) emphasizes that all patient safety incidents should be reported so that healthcare institutions could learn from their mistakes or experience.

In South Africa, reporting of adverse events is guided by the National Guidelines for Patient Safety Incidents Reporting and Learning in the Public Sector of South Africa (1). The guideline highlight that all healthcare facilities should have a Standard Operating Procedure (SOP) in place to serve as a guideline for reporting all patient safety incidents (1). The availability of the National guideline and SOP for reporting adverse events in the selected hospitals was discovered during follow-up questions in the interviews. Despite the availability of these guidelines, it was revealed that the knowledge of the professional nurses regarding the timelines of reporting, the person responsible for reporting and the tool that is used to report adverse events is differing. This finding could be due to either lack of knowledge of the contents of the available guidelines or a lack of education and training about the reporting procedure of adverse events. The professional nurses’ differing knowledge revealed in this study suggests lack of uniformity in the practice of reporting adverse events in the studied healthcare institutions Similarly, Pramesona et al. (34)’ study to explore the reasons for low reporting of patient safety incidents reported nurses’ lack of comprehension of the procedure of incident reporting and contents as one of the reasons for low reporting.

Collectively, the findings of the present study underscore the need to empower nurses with information about patient safety incidents and the reporting procedure. Empowerment is characterised by organisational structures that shape working circumstances and procedures, allowing employees to function efficiently and it is acknowledged as a key tactic for both patient safety and the prosperity of healthcare facilities (35). Kanter's theory of structural empowerment highlight that performance of an employee at work is greatly impacted by their ability to access structural empowerment, particularly, the structures of resources such as personnel and equipment to complete tasks, opportunity to learn and grow, support such as feedback from co-workers and supervisors, and information about the job and organizational vision and goals (36). Providing health care professionals with information and opportunities to be trained and educated about patient safety incidents and the reporting procedure could be one way to empower nurses, ultimately promoting effective reporting of adverse events in the studied hospitals. Effective reporting of adverse events is an essential part of quality improvement procedures that promote patient safety initiatives, system transformation, and better patient outcomes (37). When adverse events are not reported, healthcare organisations could miss opportunities to make changes that would create a safer environment because they are unaware of the potential dangers in the organisation (38). In addition, healthcare providers could miss the opportunity to learn from previous mistakes (39). Therefore, to promote patient safety initiatives the knowledge gaps identified in this study must be addressed.

## 5 Limitations

A limitation of the present study is that other healthcare providers such as medical doctors, radiographers and pharmacist were not included in the study and as such the findings of the study are limited only to the nursing profession. Another limitation could be that the study was conducted in the three selected public hospitals in Mopani district, and there is a possibility that professional nurses from other health facilities in the district and other districts in Limpopo Province might have different understanding of adverse events. Therefore, the results of the present study cannot be generalised to other hospitals in Mopani district and other districts.

## 6 Conclusion

This study highlighted significant knowledge gaps among professional nurses regarding adverse events and their reporting procedures in selected public hospitals in Mopani District. Although most participants recognized that adverse events involve harm to patients, there was confusion between adverse events and near-misses, as well as inconsistencies in understanding reporting processes, responsibilities, and timelines. Such knowledge deficits may contribute to underreporting, potentially compromising patient safety and organizational learning.

To address these challenges, it is recommended that professional nurses should be empowered to effectively report adverse events. The nursing management should develop comprehensive and continuous education programs focused on patient safety incidents, and reporting procedures. In addition, nursing management should reinforce the implementation and awareness of standard operating procedures and national guidelines of patient safety incidents reporting and learning and continuous in-service trainings. Future research should expand to include other categories of healthcare professionals and diverse healthcare settings to inform broader strategies for improving patient safety culture and reporting practices.

## Data Availability

The datasets presented in this article are not readily available because of confidentiality reasons. Requests to access the datasets should be directed to Livhuwani Muthelo, livhuwani.muthelo@ul.ac.za.
